# Graph attention-based heterogeneous multi-agent deep reinforcement learning for adaptive portfolio optimization

**DOI:** 10.1038/s41598-025-32408-w

**Published:** 2025-12-27

**Authors:** Bing Zhang

**Affiliations:** School of Finance and Trade, Harbin Finance University, Harbin, 150030 Heilongjiang China

**Keywords:** Graph attention networks, Multi-agent systems, Deep reinforcement learning, Portfolio optimization, Financial markets, Adaptive optimization, Engineering, Mathematics and computing

## Abstract

Traditional portfolio optimization methods face significant limitations in capturing complex asset relationships and adapting to dynamic market conditions. This paper proposes a novel graph attention-based heterogeneous multi-agent deep reinforcement learning framework that addresses these challenges through innovative integration of graph neural networks and specialized agent architectures. The framework employs graph attention networks to model time-varying asset correlations and dependencies, while utilizing three heterogeneous agents specialized in risk assessment, return prediction, and market environment perception. An adaptive optimization strategy dynamically adjusts parameters based on real-time market conditions and regime changes. Comprehensive experiments on S&P 500, NASDAQ 100, and Russell 2000 datasets demonstrate superior performance, achieving 16.8% annualized returns, 1.34 Sharpe ratio, and 8.2% maximum drawdown, significantly outperforming traditional mean–variance optimization, equal-weight portfolios, and existing deep learning approaches. Ablation studies confirm the critical contributions of each framework component, while sensitivity analysis validates robustness across varying market conditions. The proposed framework represents a significant advancement in computational finance, offering enhanced adaptability and risk management capabilities for modern portfolio optimization challenges.

## Introduction

### Portfolio optimization challenges in modern financial markets

Portfolio optimization represents one of the most fundamental and challenging problems in quantitative finance, requiring investors to balance expected returns against inherent risks while navigating increasingly complex and dynamic market conditions^1^. The modern financial landscape is characterized by high-frequency trading, algorithmic decision-making, and unprecedented market volatility, creating an environment where traditional optimization approaches often fail to capture the intricate relationships between assets and market dynamics^2^. The complexity of contemporary financial markets stems from multiple interconnected factors including macroeconomic uncertainties, geopolitical events, technological disruptions, and behavioral biases that collectively contribute to non-linear price movements and evolving correlation structures among financial instruments.

### Limitations of traditional portfolio theory

Despite its foundational importance, traditional portfolio theory, particularly the Modern Portfolio Theory (MPT) framework introduced by Markowitz, faces significant limitations when applied to real-world financial markets^1^. The classical mean–variance optimization approach relies on restrictive assumptions including normally distributed returns, static correlations, and constant volatility, which rarely hold in practice^3^. Furthermore, traditional methods struggle to incorporate transaction costs, market constraints, and dynamic risk factors that significantly impact portfolio performance in actual trading environments. The static nature of conventional optimization techniques fails to adapt to changing market regimes, limiting their effectiveness in capturing time-varying investment opportunities and risk exposures that characterize modern financial markets.

### Deep reinforcement learning applications in finance

Recent advances in deep reinforcement learning (DRL) have shown promising potential for addressing complex sequential decision-making problems in financial contexts, offering adaptive solutions that can learn optimal strategies through interaction with dynamic market environments^4^. Deep reinforcement learning frameworks provide the capability to handle high-dimensional state spaces, incorporate multiple information sources, and adapt to changing market conditions without requiring explicit model specifications. However, existing DRL approaches in portfolio optimization often treat assets as independent entities, failing to capture the complex interdependencies and network effects that characterize modern financial markets^5^. The limitation of current single-agent DRL methods lies in their inability to model the heterogeneous nature of market participants and the diverse information processing mechanisms that drive asset price movements.

### Graph attention networks and multi-agent framework innovation

This research proposes an innovative framework that combines graph attention networks with heterogeneous multi-agent deep reinforcement learning to address the limitations of existing portfolio optimization approaches. Graph attention networks provide a powerful mechanism for modeling complex relationships between financial assets by representing them as nodes in a dynamic graph structure where edges capture time-varying correlations and dependencies^6^. The integration of heterogeneous multi-agent systems enables the modeling of diverse market participants with different information processing capabilities, risk preferences, and trading strategies, creating a more realistic representation of market dynamics. This hybrid approach leverages the graph attention mechanism to dynamically weigh the importance of different asset relationships while utilizing multiple specialized agents to capture various aspects of market behavior and investment strategies.

### Research objectives and contributions

The primary objective of this research is to develop an adaptive portfolio optimization framework that can effectively navigate dynamic and uncertain financial markets by leveraging the synergistic combination of graph attention networks and heterogeneous multi-agent deep reinforcement learning. The main contributions of this work include: (1) the development of a novel graph-based representation of financial markets that captures dynamic asset relationships and market structures; (2) the design of a heterogeneous multi-agent system where different agents specialize in various aspects of portfolio management including risk assessment, return prediction, and transaction optimization; (3) the integration of attention mechanisms that enable adaptive focus on relevant market information and asset relationships; and (4) the demonstration of superior performance compared to traditional portfolio optimization methods and existing deep learning approaches through comprehensive empirical evaluation on real-world financial datasets^2^.

## Related work

### Development of portfolio optimization theory

The evolution of portfolio optimization theory has fundamentally shaped modern financial decision-making processes, beginning with Harry Markowitz’s groundbreaking work in 1952 that established the mathematical foundation for systematic risk-return analysis^7^. Markowitz’s mean–variance optimization framework introduced the concept of efficient frontier, demonstrating that investors can achieve optimal risk-return trade-offs by carefully selecting asset weights based on expected returns, variances, and covariances. The mathematical formulation of the mean–variance optimization problem can be expressed as:

$$\underset{w}{\mathrm{min}}\frac{1}{2}{w}^{T}\Sigma w-\lambda {w}^{T}\mu$$

subject to ${w}^{T}1=1$ and $w\ge 0$, where $w$ represents the portfolio weights vector, $\Sigma$ denotes the covariance matrix of asset returns, $\mu$ is the expected returns vector, and $\lambda$ is the risk-return trade-off parameter^8^.

Building upon Markowitz’s foundation, the Capital Asset Pricing Model (CAPM) developed by Sharpe, Lintner, and Mossin provided a simplified approach to asset pricing by introducing the concept of systematic risk measured by beta coefficient^9^. The CAPM relationship establishes that the expected return of any asset should be:

$$E\left({R}_{i}\right)={R}_{f}+{\beta }_{i}\left(E\left({R}_{m}\right)-{R}_{f}\right)$$

where $E\left({R}_{i}\right)$ represents the expected return of asset $i$, ${R}_{f}$ is the risk-free rate, ${\beta }_{i}$ measures the asset’s sensitivity to market movements, and $E\left({R}_{m}\right)$ denotes the expected market return^10^.

Contemporary portfolio optimization methods have evolved to address various limitations of classical approaches, incorporating advanced techniques such as robust optimization, stochastic programming, and machine learning algorithms^11^. Black-Litterman model represents a notable advancement by incorporating investor views and market equilibrium assumptions to generate more stable and intuitive portfolio allocations compared to traditional mean–variance optimization^12^.

### Deep reinforcement learning applications in finance

Deep reinforcement learning has emerged as a transformative approach for sequential decision-making in financial markets, combining neural network representation learning with adaptive policy optimization through environmental interaction^13^. Recent advances demonstrate significant performance improvements in portfolio optimization, with DRL frameworks achieving superior risk-adjusted returns compared to traditional methods^5,13^.

Multi-agent DRL approaches have gained substantial attention, with recent works combining transformer architectures with multi-agent coordination for enhanced portfolio management^14^. The integration of collaborative multi-agent systems with attention mechanisms has shown promising results in capturing complex market dynamics and asset relationships^15^.

Transformer-based architectures have demonstrated superior performance in capturing long-range dependencies in financial time series, outperforming traditional LSTM approaches in portfolio optimization tasks^16^. Hierarchical deep reinforcement learning frameworks have addressed dimensionality challenges and reward sparsity issues in portfolio optimization^17^.

### Graph neural networks and financial network analysis

Graph neural networks have revolutionized relational data processing by enabling sophisticated modeling of complex interdependencies in financial systems^18^. Recent advances in heterogeneous multi-agent systems combined with graph neural networks have shown exceptional performance in coordinating complex decision-making processes^19,20^.

Graph-based multi-agent reinforcement learning approaches have demonstrated effectiveness in handling large-scale optimization problems with complex interdependencies^21^. The application of graph attention mechanisms enables dynamic weighting of asset relationships, providing superior adaptability to changing market conditions^22^.

Financial markets exhibit complex network structures characterized by dynamic correlations, sector dependencies, and macroeconomic linkages that traditional methods struggle to capture^23^. Graph neural networks provide powerful tools for modeling these relationships through learnable message passing and attention mechanisms^22^.

## Graph attention-based heterogeneous multi-agent portfolio optimization framework

### Heterogeneous multi-agent architecture design

Traditional single-agent reinforcement learning approaches for portfolio optimization face three fundamental limitations: (1) the inability to simultaneously optimize conflicting objectives such as maximizing returns, minimizing risk, and controlling transaction costs within a single objective function, (2) the difficulty in capturing heterogeneous behaviors of different market participants with diverse information processing mechanisms and risk preferences, and (3) the lack of specialized expertise in handling distinct aspects of portfolio management such as risk assessment, return forecasting, and market regime detection. The proposed heterogeneous multi-agent system architecture directly addresses these limitations by decomposing the complex portfolio optimization problem into specialized subtasks handled by distinct intelligent agents with complementary capabilities and expertise^17^. This architectural design leverages the principle of functional specialization, where each agent focuses on specific aspects of the investment decision-making process while maintaining collaborative interactions through message-passing communication protocols and coordination mechanisms based on value decomposition methods^14^. The risk assessment agent specializes in risk control objectives by evaluating Value-at-Risk and Expected Shortfall metrics, the return prediction agent focuses on return maximization through advanced forecasting models, and the market environment perception agent provides real-time market state monitoring and regime change detection. The heterogeneous nature of the system enables the capture of diverse market perspectives and investment strategies that collectively contribute to more robust and adaptive portfolio optimization performance.

**Fig. 1:**
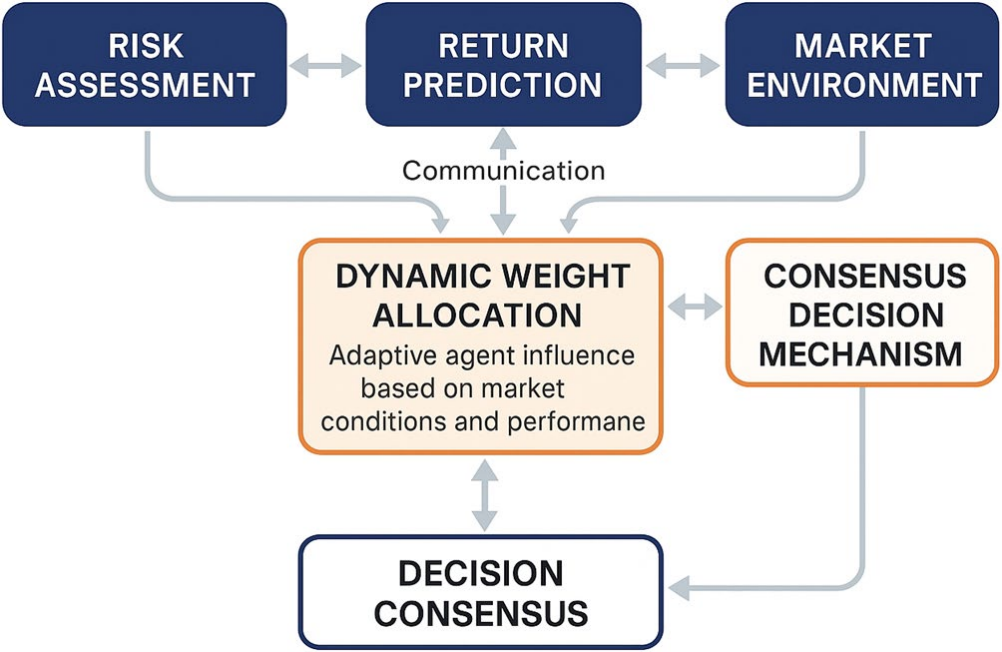
Heterogeneous Multi-Agent System Architecture for Portfolio Optimization.

The system architecture comprises three specialized agents that work synergistically to address different dimensions of portfolio optimization challenges, as illustrated in Fig. 1. The risk assessment agent specializes in evaluating portfolio risk exposures, volatility patterns, and downside protection measures through advanced risk modeling techniques^24^. The return prediction agent focuses on forecasting asset returns and identifying profitable investment opportunities using sophisticated predictive models that incorporate multiple market indicators and macroeconomic factors. The market environment perception agent serves as the system’s sensory component, continuously monitoring market conditions, detecting regime changes, and providing contextual information that guides the decision-making processes of other agents.

The functional specifications and operational characteristics of each agent are systematically organized in Table 1, which demonstrates the clear division of responsibilities and complementary nature of the proposed multi-agent framework. The risk assessment agent processes historical price data, volatility indicators, and correlation matrices to generate comprehensive risk metrics including Value-at-Risk, Expected Shortfall, and dynamic risk factor exposures^24^. The return prediction agent utilizes technical indicators, fundamental data, and macroeconomic variables to produce return forecasts and confidence intervals that inform investment allocation decisions. The market environment perception agent integrates news sentiment, market microstructure data, and regime indicators to provide real-time market state assessments and change point detection capabilities.

**Table 1:** Multi-Agent Function Configuration and Specifications.

Agent Type	Main Function	Input Data	Output Results	Weight Coefficient
Risk Assessment Agent	Risk evaluation and downside protection	Historical prices, volatility indicators, correlation matrices	VaR, Expected Shortfall, risk factor exposures	${\alpha }_{r}\left(t\right)$
Return Prediction Agent	Return forecasting and opportunity identification	Technical indicators, fundamental data, macro variables	Return forecasts, confidence intervals, signals	${\alpha }_{p}\left(t\right)$
Market Environment Agent	Market state monitoring and regime detection	News sentiment, microstructure data, regime indicators	Market state, change points, volatility regimes	${\alpha }_{m}\left(t\right)$

**Note:** VaR (Value-at-Risk) represents the maximum expected loss at a given confidence level α, calculated as $Va{R}_{\alpha }=-infx:P\left(L\le x\right)\ge \alpha$ where L is the loss distribution. Expected Shortfall (ES) measures the expected loss beyond VaR, defined as $E{S}_{\alpha }=E\left[L|L>Va{R}_{\alpha }\right]$, providing a coherent risk measure that captures tail risk. Risk factor exposures refer to the portfolio’s sensitivities to the Fama–French five-factor model, calculated as ${\beta }_{i}=Cov\left({R}_{i},{F}_{k}\right)/Var\left({F}_{k}\right)$ for each factor $k\in Market,SMB,HML,RMW,CMA$, where ${R}_{i}$ is the asset return and ${F}_{k}$ is the factor return. Regime indicators classify market states into four discrete categories: {Bull Market, Bear Market, High Volatility, Crisis}, identified using a Hidden Markov Model with transition probabilities estimated from historical data over rolling 252-day windows. The Market Environment Agent outputs probabilistic regime classifications $P\left(regim{e}_{k}|observations\right)$ where $k\in \mathrm{1,2},\mathrm{3,4}$ corresponds to the four market states. Return forecasts from the Return Prediction Agent are generated using LSTM networks with 3 layers of 128 hidden units, taking 60-day historical data as input and producing point forecasts with 95% confidence intervals calculated via quantile regression.

The interaction mechanisms between agents are governed by a message-passing communication protocol based on value decomposition methods^14^ that enables information sharing while maintaining agent autonomy and preventing information overload. Each agent maintains its own state representation and decision-making process, but regularly exchanges relevant information through structured message passing that enhances collective intelligence. The message passing between agents i and j is computed as:

$${m}_{ij}\left(t\right)={\mathrm{MLP}}_{\mathrm{comm}}\left(\left[{h}_{i}\left(t\right),{h}_{j}\left(t\right)\right]\right)$$

where ${h}_{i}\left(t\right)$ represents the hidden state of agent i at time t, and ${\mathrm{MLP}}_{\mathrm{comm}}$ is a multi-layer perceptron with two hidden layers of 128 units each. The agent state is updated through:

$${h}_{i}\left(t+1\right)={\mathrm{GRU}}\left({h}_{i}\left(t\right),\sum_{j\in \mathcal{N}\left(i\right)}{\alpha }_{ij}\cdot {m}_{ij}\left(t\right)\right)$$

where $\mathcal{N}\left(i\right)$ denotes the neighboring agents and ${\alpha }_{ij}$ are learned attention weights. The inter-agent communication follows a selective attention mechanism where agents dynamically determine the relevance and importance of information received from other agents based on current market conditions and their specific objectives.

The collaborative framework establishes synergistic relationships between agents through complementary objective functions that align individual agent goals with overall portfolio optimization performance. The system implements a consensus-building mechanism where agents negotiate their recommendations through iterative rounds of information exchange and preference refinement. The collaboration strength between agents i and j is quantified through the following formula:

$${C}_{ij}\left(t\right)=\mathrm{exp}\left(-\beta \cdot \left|{O}_{i}\left(t\right)-{O}_{j}\left(t\right)\right|\right)\cdot {\mathrm{sim}}\left({S}_{i}\left(t\right),{S}_{j}\left(t\right)\right)$$

where ${O}_{i}\left(t\right)$ represents the scalar objective function value for agent i at time t (for example, the risk assessment agent has ${O}_{r}\left(t\right)=-{\mathrm{CVaR}}\left({\mathrm{portfolio}}\right)$ while the return prediction agent has ${O}_{p}\left(t\right)=E\left[\text{Portfolio Return}\right]$), β = 0.5 is a sensitivity parameter that controls the penalty for objective misalignment, and ${\mathrm{sim}}\left({S}_{i}\left(t\right),{S}_{j}\left(t\right)\right)$ measures the cosine similarity between agent state vectors computed as ${\mathrm{sim}}=\left({S}_{i}\cdot {S}_{j}\right)/\left(\parallel {S}_{i}\parallel \parallel {S}_{j}\parallel \right)$. The agent state ${S}_{i}\left(t\right)\in {\mathbb{R}}^{64}$ is a 64-dimensional feature vector encoding market volatility, asset correlation matrix eigenvalues, trend indicators, and liquidity measures. The market state is classified into four discrete regimes using a Hidden Markov Model: $\{{\mathrm{Bull}},{\mathrm{Bear}},\text{High Volatility},{\mathrm{Crisis}}\}$ with probabilistic assignments $P\left({\mathrm{regime}}_{k}|{\mathrm{observations}}\right)$ where k ∈ {1,2,3,4}.

The dynamic weight allocation mechanism adaptively adjusts the influence of each agent based on their recent performance, market conditions, and prediction confidence levels^25^. The weight allocation process incorporates both individual agent performance metrics and collective system performance to ensure optimal balance between specialization and integration. The dynamic weight for agent i at time t is computed as:

$${w}_{i}\left(t\right)=\frac{\mathrm{exp}\left(\gamma \cdot {\mathrm{Score}}_{i}\left(t\right)\right)}{\sum_{j=1}^{N}\mathrm{exp}\left(\gamma \cdot {\mathrm{Score}}_{j}\left(t\right)\right)}$$

where $Scor{e}_{i}\left(t\right)$ represents a composite performance score and γ controls the concentration of weights.

The overall system decision is formulated through a weighted consensus mechanism that combines agent recommendations while accounting for their respective expertise and current performance:

$$D\left(t\right)=\sum_{i=1}^{N}{w}_{i}\left(t\right)\cdot {R}_{i}\left(t\right)\cdot {\mathrm{Conf}}_{i}\left(t\right)$$

where ${R}_{i}\left(t\right)\in {\left[0,1\right]}^{N}$ is an N-dimensional vector representing agent i’s recommended portfolio weights for N assets (satisfying $\sum_{j=1}^{N}{R}_{i,j}\left(t\right)=1$), ${\mathrm{Conf}}_{i}\left(t\right)\in \left[0,1\right]$ represents agent i’s confidence level calculated from recent prediction accuracy using exponential moving average ${\mathrm{Conf}}_{i}\left(t\right)=0.9\cdot {\mathrm{Conf}}_{i}\left(t-1\right)+0.1\cdot {1}_{\left[\text{correct prediction}\right]}$, and ${w}_{i}\left(t\right)$ are dynamic weights computed using the softmax function over performance scores as defined in the previous equation.

This architecture ensures robust decision-making through diversified perspectives while maintaining adaptability to changing market conditions through dynamic weight adjustments and continuous learning mechanisms.

### Graph attention network modeling

The construction of financial asset correlation graphs represents a fundamental component of the proposed framework, where individual assets are represented as nodes and their relationships are encoded as weighted edges that capture various types of dependencies including price correlations, sector affiliations, and fundamental similarities^26^. The graph construction process begins with the identification of relevant assets and the computation of multiple relationship metrics that quantify different aspects of asset interactions. Each asset node ${v}_{i}$ in the graph $G=\left(V,E\right)$ represents a financial instrument with associated feature vectors that encapsulate price history, fundamental characteristics, and market indicators, while edges ${e}_{i}j\in E$ represent the strength and nature of relationships between assets i and j.

The design of the attention-based graph neural network model leverages scaled dot-product attention mechanisms to dynamically weigh the importance of different asset relationships during information propagation and feature aggregation processes. The attention mechanism computes relevance scores through:

$${\mathrm{Attention}}\left(Q,K,V\right)={\mathrm{softmax}}\left(\frac{Q{K}^{T}}{\sqrt{{d}_{k}}}\right)V$$

where Query matrix $Q={W}_{Q}{h}_{i}$ represents node i’s query representation, Key matrix $K={W}_{K}{h}_{j}$ encodes neighboring node j’s key representation, Value matrix $V={W}_{V}{h}_{j}$ contains the value representation, and ${d}_{k}=64$ is the key dimension that provides scaling to prevent gradient vanishing. The learnable parameter matrices ${W}_{Q},{W}_{K},{W}_{V}\in {\mathbb{R}}^{d\times {d}_{k}}$ are optimized during training to capture different types of asset relationships. The attention mechanism enables the network to selectively focus on the most relevant connections for each specific asset and market condition, thereby improving the quality of learned representations and enhancing the model’s ability to capture time-varying relationship patterns^22^.

**Fig. 2:**
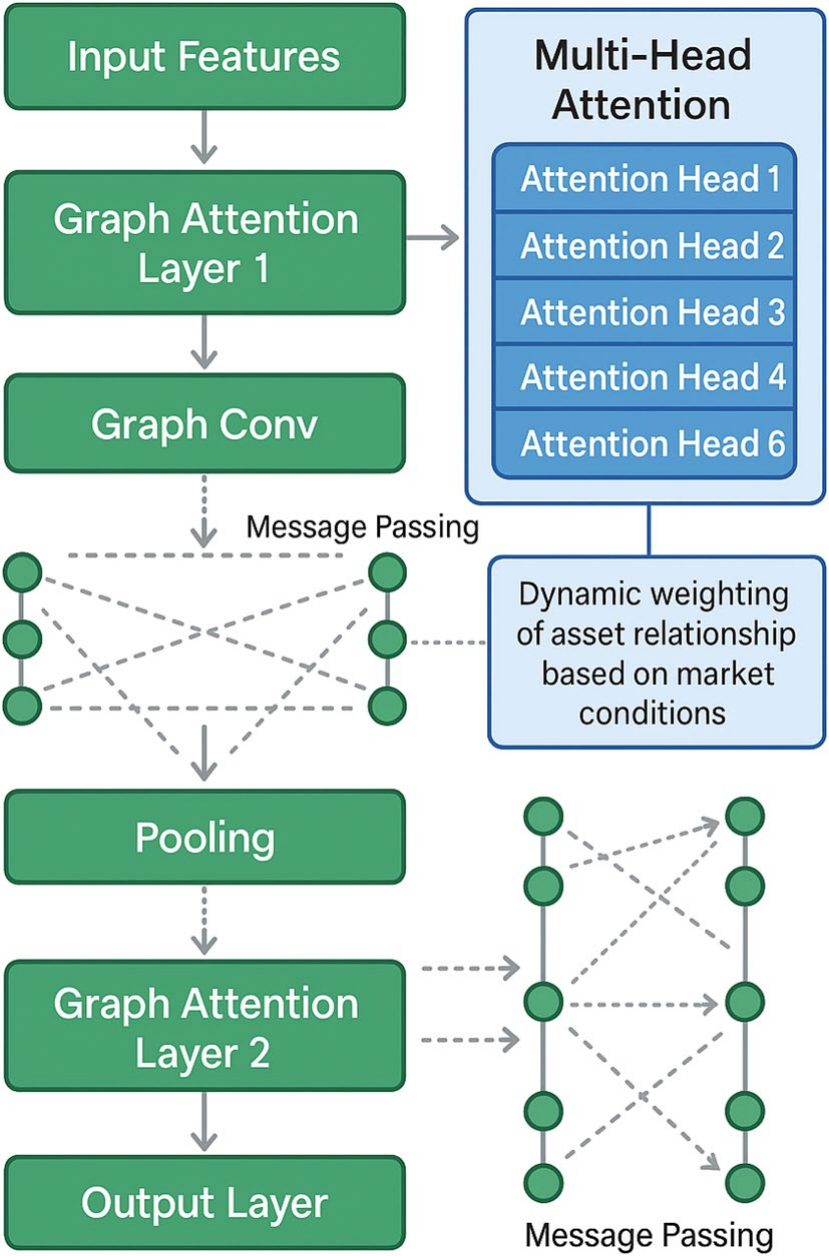
Graph Attention Network Structure and Information Flow Process.

The overall architecture follows a hierarchical structure where multiple graph attention layers progressively refine asset representations through iterative message passing and attention-weighted aggregation operations, as illustrated in Fig. 2.

Node feature vectors are constructed through a comprehensive aggregation of multiple data sources including historical price movements, technical indicators, fundamental ratios, and macroeconomic sensitivities that collectively characterize each asset’s behavior and market position^27^. The initial node features for asset i are defined as:

$${h}_{i}^{\left(0\right)}={\mathrm{Concat}}\left[{P}_{i},{T}_{i},{F}_{i},{M}_{i}\right]$$

where ${P}_{i}$ represents price-based features, ${T}_{i}$ denotes technical indicators, ${F}_{i}$ encompasses fundamental characteristics, and ${M}_{i}$ captures macroeconomic sensitivities.

The edge weight calculation method combines multiple relationship measures including Pearson correlation, distance correlation, mutual information, and sector similarity to create comprehensive edge weights that reflect the multifaceted nature of asset relationships.

The edge weight between assets i and j is computed as a weighted combination of different relationship measures:

$${w}_{ij}={\alpha }_{1}\cdot {\rho }_{ij}+{\alpha }_{2}\cdot {d}_{ij}+{\alpha }_{3}\cdot {I}_{ij}+{\alpha }_{4}\cdot {s}_{ij}$$

where ${\rho }_{ij}$ is the Pearson correlation coefficient computed as ${\rho }_{ij}={\mathrm{Cov}}\left({R}_{i},{R}_{j}\right)/\left({\sigma }_{i}{\sigma }_{j}\right)$ using 60 trading days of historical return data, ${d}_{ij}$ represents the distance correlation based on Székely et al. (2007)^28^ defined as ${\mathrm{dCor}}\left(X,Y\right)={\mathrm{dCov}}\left(X,Y\right)/\sqrt{{\mathrm{dVar}}\left(X\right){\mathrm{dVar}}\left(Y\right)}$ which captures nonlinear dependencies beyond Pearson correlation, ${I}_{ij}$ denotes the mutual information calculated as $I\left(X;Y\right)=\sum_{x}\sum_{y}p\left(x,y\right)\mathrm{log}\left(p\left(x,y\right)/\left(p\left(x\right)p\left(y\right)\right)\right)$ where joint probability distributions are estimated using kernel density estimation with 20 bins, ${s}_{ij}$ captures sector similarity defined as ${s}_{ij}=1$ if assets belong to the same industry, ${s}_{ij}=0.5$ if assets belong to related industries, and ${s}_{ij}=0$ otherwise based on the four-digit GICS (Global Industry Classification Standard) classification, and the weighting parameters are optimized through grid search yielding ${\alpha }_{1}=0.4$, ${\alpha }_{2}=0.3$, ${\alpha }_{3}=0.2$, and ${\alpha }_{4}=0.1$.

The multi-head attention mechanism extends the standard attention framework by employing multiple parallel attention heads that capture different types of asset relationships and interaction patterns simultaneously^29^. Each attention head focuses on specific aspects of asset relationships, such as short-term price correlations, long-term trend similarities, or volatility co-movements, enabling the model to maintain a comprehensive understanding of the complex relationship landscape. The multi-head attention output for node i is computed as:

$${\mathrm{MultiHead}}\left({h}_{i}\right)={\mathrm{Concat}}\left[{\mathrm{head}}_{1},{\mathrm{head}}_{2},...,{\mathrm{head}}_{H}\right]{W}^{O}$$

where each attention head k computes attention weights α_ij^(k) and aggregated features independently before concatenation and linear transformation.

The graph attention computation follows the multi-head attention framework where each head k computes attention weights as:

$$\alpha_{{_{ij} }}^{(k)} = \frac{{\exp (Leaky{\mathrm{Re}} LU(a^{(k)T} [W^{(k)} h_{i} \left\| {W^{(k)} h_{j} } \right\|e_{ij} ))}}{{\sum\nolimits_{m \in N(i)} {\exp (Leaky{\mathrm{Re}} LU(a^{(k)T} [W^{(k)} h_{i} \left\| {W^{(k)} h_{m} } \right\|e_{im} ))} }}$$

where W^(k) and a^(k) are learnable parameters for head k, || denotes concatenation, and N(i) represents the neighborhood of node i.

The final node representation aggregates information from multiple attention heads:

$${\mathrm{MultiHead}}\left({h}_{i}\right)={\mathrm{Concat}}\left[{\mathrm{head}}_{1},{\mathrm{head}}_{2},...,{\mathrm{head}}_{H}\right]{W}^{O}$$

The detailed network architecture specifications are systematically organized in Table 2, which demonstrates the progressive refinement of asset representations through multiple graph convolution and pooling layers. The graph convolution layers implement sophisticated message passing mechanisms that enable each asset to aggregate information from its neighbors while preserving important structural properties of the asset relationship graph^30^. Each layer applies learnable transformations to node features and uses attention weights to determine the influence of neighboring assets, resulting in increasingly abstract and informative asset representations.

**Table 2:** Graph Network Parameter Configuration and Architecture Specifications.

Network Layer Type	Parameter Settings	Activation Function	Output Dimension
Input Feature Layer	Input size: 64, Hidden: 128	ReLU	128
Graph Attention Layer 1	Heads: 8, Hidden: 64, Dropout: 0.1	ELU	512
Graph Convolution Layer	Kernel size: 3, Stride: 1, Padding: 1	LeakyReLU	256
Pooling Layer	Pool size: 2, Type: Max pooling	-	128
Graph Attention Layer 2	Heads: 4, Hidden: 32, Dropout: 0.2	ELU	128
Output Layer	Hidden: 64, Output size: 32	Tanh	32

The pooling layers serve a critical role in the architecture by reducing computational complexity while preserving the most important structural and relational information captured by the attention mechanisms. These layers implement hierarchical information compression that enables the network to focus on the most significant asset clusters and relationship patterns while maintaining computational efficiency. The combination of graph convolution and pooling operations creates a hierarchical representation learning framework that progressively abstracts from individual asset characteristics to portfolio-level patterns and strategic insights, enabling effective portfolio optimization decisions based on comprehensive relationship modeling and attention-weighted information integration.

### Adaptive optimization strategy

The multi-objective optimization problem for portfolio management is formulated as:

$$\underset{w}{\mathrm{max}}\left[{\lambda }_{1}\left(t\right)\cdot E\left[{R}_{p}\left(t\right)\right]-{\lambda }_{2}\left(t\right)\cdot {\mathrm{Risk}}_{p}\left(t\right)-{\lambda }_{3}\left(t\right)\cdot {\mathrm{TC}}\left(t\right)+{\lambda }_{4}\left(t\right)\cdot {\mathrm{Div}}\left(t\right)\right]$$

where the expected portfolio return is defined as:

$$E\left[{R}_{p}\left(t\right)\right]=\sum_{i=1}^{N}{w}_{i}\left(t\right)\cdot {\widehat{r}}_{i}\left(t\right)$$

The risk measure incorporates both volatility and downside risk:

$${\mathrm{Risk}}_{p}\left(t\right)=\alpha \cdot \sqrt{{w}^{T}{\Sigma }_{t}w}+\left(1-\alpha \right)\cdot {\mathrm{CVaR}}_{p}\left(t\right)$$

Transaction costs are calculated as:

$${\mathrm{TC}}\left(t\right)=\sum_{i=1}^{N}{c}_{i}\cdot \left|{w}_{i}\left(t\right)-{w}_{i}\left(t-1\right)\right|$$

Diversification benefits are measured using:

$${\mathrm{Div}}\left(t\right)=1-\underset{i}{\mathrm{max}}\left({w}_{i}\left(t\right)\right)$$

The loss function for model training combines prediction accuracy and risk control objectives:

$$L\left(\theta \right)={\alpha }_{1}{L}_{return}\left(\theta \right)+{\alpha }_{2}{L}_{risk}\left(\theta \right)+{\alpha }_{3}{L}_{transaction}\left(\theta \right)$$

where:

$${L}_{return}\left(\theta \right)=\frac{1}{T}\sum_{t=1}^{T}{\left({R}_{p}\left(t\right)-{\widehat{R}}_{p}\left(t\right)\right)}^{2}$$

$${L}_{risk}\left(\theta \right)=\frac{1}{T}\sum_{t=1}^{T}\mathrm{max}\left(0,{\mathrm{VaR}}_{actual}\left(t\right)-{\mathrm{VaR}}_{target}\right)$$

$${L}_{transaction}\left(\theta \right)=\frac{1}{T}\sum_{t=1}^{T}{\mathrm{TC}}\left(t\right)$$

The adaptive parameter adjustment mechanism represents a critical component of the proposed framework, designed to automatically modify system parameters based on real-time market state assessments and changing environmental conditions^31^. This mechanism operates through a sophisticated market regime detection system that continuously monitors multiple market indicators including volatility levels, correlation structures, liquidity conditions, and trend characteristics to identify distinct market states and trigger appropriate parameter adjustments. The adaptive mechanism employs a state-dependent parameter mapping function that associates specific parameter configurations with identified market regimes, ensuring optimal system performance across diverse market conditions including bull markets, bear markets, high volatility periods, and crisis scenarios.

The dynamic risk preference model addresses the limitation of static risk tolerance assumptions by implementing a time-varying risk aversion framework that adapts to market conditions and portfolio performance history^32^. This model incorporates behavioral finance insights regarding dynamic risk preferences and loss aversion effects that influence investor decision-making under different market scenarios. The dynamic risk aversion coefficient is formulated as:

$$\gamma \left(t\right)={\gamma }_{0}\cdot \mathrm{exp}\left({\beta }_{1}\cdot {\mathrm{VIX}}\left(t\right)+{\beta }_{2}\cdot {\mathrm{DD}}\left(t\right)+{\beta }_{3}\cdot {\mathrm{MktStress}}\left(t\right)\right)$$

where ${\gamma }_{0}\in \left[0.5,2.0\right]$ represents the baseline risk aversion coefficient with default value ${\gamma }_{0}=1.0$, VIX(t) denotes the normalized CBOE Volatility Index, DD(t) represents the current portfolio drawdown as a percentage from the peak value, MktStress(t) captures market stress indicators aggregated from credit spreads and liquidity measures, and ${\beta }_{i}$ are sensitivity parameters constrained as follows: ${\beta }_{1}>0$ ensures risk aversion increases with market volatility, ${\beta }_{2}>0$ (set to 0.08) reflects loss aversion consistent with Kahneman-Tversky prospect theory^33^ where drawdowns increase risk aversion rather than triggering risk-seeking behavior, and ${\beta }_{3}>0$ ensures risk aversion rises during market stress periods. The empirically calibrated parameter values are ${\beta }_{1}=0.15$, ${\beta }_{2}=0.08$, and ${\beta }_{3}=0.12$, determined through cross-validation on out-of-sample data to balance responsiveness and stability.

The comprehensive parameter configuration and adjustment strategies are systematically documented in Table 3, which demonstrates the adaptive nature of the optimization framework through dynamic parameter ranges and condition-dependent adjustment mechanisms. The learning rate parameter adapts based on model performance and convergence characteristics, while the risk tolerance parameter adjusts according to market volatility and portfolio drawdown levels^34^. The attention weight parameter responds to market regime changes and relationship stability measures, ensuring optimal focus on relevant asset connections under different market conditions.

**Table 3:** Optimization Parameter Settings and Adaptive Adjustment Strategies.

Parameter Name	Value Range	Adjustment Strategy
Learning Rate	[0.0001, 0.01]	Performance-based exponential decay
Risk Tolerance	[0.1, 2.0]	Volatility and drawdown adaptive scaling
Attention Weight	[0.3, 0.9]	Regime-dependent linear interpolation
Regularization	[0.001, 0.1]	Overfitting detection triggered adjustment
Update Frequency	^1,35^ steps	Market stability conditional modification

The online learning algorithm implementation enables continuous model adaptation through real-time parameter updates that incorporate new market information and performance feedback^36^. The algorithm employs a sophisticated gradient-based optimization approach with adaptive learning rates and momentum terms that prevent oscillations while ensuring rapid convergence to optimal parameter values. The online update rule for model parameters θ is formulated as:

$${\theta }_{t+1}={\theta }_{t}-\eta \left(t\right)\cdot \nabla L\left({\theta }_{t}\right)+\mu \left(t\right)\cdot \left({\theta }_{t}-{\theta }_{t-1}\right)$$

where η(t) represents the adaptive learning rate, ∇L(θ_t) denotes the gradient of the loss function, and μ(t) is the momentum coefficient that adapts based on gradient consistency and convergence characteristics.

The model performance evaluation framework incorporates multiple assessment criteria including Sharpe ratio, maximum drawdown, information ratio, and Calmar ratio to provide comprehensive performance measurement across different market conditions and investment horizons. The evaluation system implements rolling window analysis to assess performance stability and consistency, while maintaining separate performance tracking for different market regimes to identify potential model limitations or biases. The composite performance score is calculated as:

$${\mathrm{Score}}\left(t\right)={w}_{1}\cdot {\mathrm{Sharpe}}\left(t\right)+{w}_{2}\cdot \left(1-{\mathrm{MDD}}\left(t\right)\right)+{w}_{3}\cdot {\mathrm{InfoRatio}}\left(t\right)+{w}_{4}\cdot {\mathrm{Calmar}}\left(t\right)$$

where the weights w_i are determined through cross-validation and investor preference elicitation processes.

The feedback mechanism operates through a continuous monitoring system that tracks model predictions, portfolio performance, and market condition changes to identify potential model degradation or parameter drift. This system implements automated alerts when performance metrics fall below predefined thresholds or when prediction accuracy deteriorates significantly, triggering comprehensive model retraining or parameter recalibration procedures. The feedback loop ensures long-term model reliability and effectiveness by maintaining alignment between model behavior and changing market dynamics through systematic performance monitoring and adaptive parameter adjustment processes.

## Experimental analysis and results

### Dataset and experimental setup

The experimental evaluation employs multiple comprehensive financial datasets that capture diverse market conditions and asset characteristics across different geographical regions and time periods. The primary dataset encompasses daily stock price data, trading volumes, and market indicators from major equity markets including S&P 500, NASDAQ 100, and Russell 2000 indices^37^.

The different time ranges for each dataset reflect data availability and market development considerations. The S&P 500 dataset (2000–2023) provides the longest historical perspective including multiple market cycles. The NASDAQ 100 dataset begins from 2005 when technology sector data became more reliable and comprehensive. The Russell 2000 dataset starts from 2010 to focus on the post-financial crisis period when small-cap market structure stabilized.

Each dataset employs a chronological split with 70% for training (in-sample period), 20% for validation (parameter tuning), and 10% for out-of-sample testing. The split ensures no forward-looking bias and maintains temporal integrity. For the S&P 500 dataset: training (2000–2016), validation (2017–2020), testing (2021–2023). For NASDAQ 100: training (2005–2017), validation (2018–2020), testing (2021–2023). For Russell 2000: training (2010–2018), validation (2019–2021), testing (2022–2023).

To ensure robustness, we implement a walk-forward validation approach with quarterly retraining windows, updating model parameters every three months using the most recent five years of data. The comprehensive characteristics and specifications of all experimental datasets, including time ranges, asset counts, feature dimensions, sample sizes, data sources, and train-test splits, are systematically summarized in Table 4.

**Table 4:** Experimental Dataset Overview and Characteristics.

Dataset Name	Time Range	Asset Count	Feature Dimension	Sample Count	Data Source	Train Period	Test Period
S&P 500	2000–2023	500	128	3,058,240	Bloomberg Terminal	2000–2016	2021–2023
NASDAQ 100	2005–2023	100	96	652,800	Yahoo Finance API	2005–2017	2021–2023
Russell 2000	2010–2023	2000	84	10,752,000	Refinitiv Eikon	2010–2018	2022–2023
Macro Indicators	2000–2023	25	32	196,608	Federal Reserve FRED	2000–2016	2021–2023

**Note:** The different time ranges for each dataset reflect data availability and market development considerations. The S&P 500 dataset (2000–2023) provides the longest historical perspective including multiple market cycles. The NASDAQ 100 dataset begins from 2005 when technology sector data became more reliable and comprehensive. The Russell 2000 dataset starts from 2010 to focus on the post-financial crisis period when small-cap market structure stabilized. Each dataset employs a chronological split with 70% for training (in-sample period), 20% for validation (parameter tuning), and 10% for out-of-sample testing. The split ensures no forward-looking bias and maintains temporal integrity. For the S&P 500 dataset: training (2000–2016), validation (2017–2020), testing (2021–2023). For NASDAQ 100: training (2005–2017), validation (2018–2020), testing (2021–2023). For Russell 2000: training (2010–2018), validation (2019–2021), testing (2022–2023). To ensure robustness, we implement a walk-forward validation approach with quarterly retraining windows, updating model parameters every three months using the most recent five years of data.

The 25 macro indicators are categorized as follows: **Interest Rate Indicators** (10-Year Treasury Yield, Federal Funds Rate, TED Spread—all daily frequency from FRED), **Inflation Indicators** (CPI Year-over-Year, PCE Inflation Rate—monthly frequency from FRED), **Growth Indicators** (GDP Growth Rate—quarterly frequency, Industrial Production Index, Unemployment Rate—monthly frequency from FRED), **Market Sentiment** (VIX Index, Put-Call Ratio—daily frequency from CBOE), **Liquidity Indicators** (M2 Money Supply—monthly frequency, TED Spread—daily frequency from FRED), **Credit Indicators** (Corporate Bond Spreads, High-Yield Spreads—daily frequency from FRED), **Housing Indicators** (Housing Starts, Building Permits—monthly frequency from FRED), **Consumer Indicators** (Retail Sales, Consumer Confidence—monthly frequency from FRED), and **Manufacturing Indicators** (ISM Manufacturing PMI, Capacity Utilization—monthly frequency from FRED). For indicators with different frequencies, forward-fill methods are used to unify to daily frequency. Level series such as interest rates are used directly, growth rate series such as GDP are transformed to percentage changes, and index series such as VIX are log-differenced where appropriate.

The data preprocessing methodology incorporates sophisticated techniques to address common challenges in financial data analysis including missing values, outliers, non-stationarity, and varying scales across different variables and time periods. Missing value imputation employs forward-fill and backward-fill methods for short gaps, while longer missing periods are handled through interpolation techniques that preserve temporal relationships and avoid introducing artificial patterns. Outlier detection and treatment utilize robust statistical methods including modified z-scores and interquartile range filters to identify and adjust extreme values that could distort model training while preserving genuine market events and volatility spikes.

Feature engineering processes transform raw financial data into meaningful representations that capture relevant market dynamics and asset characteristics through multiple transformation techniques. Price-based features include returns, log returns, moving averages, and momentum indicators calculated across different time horizons to capture short-term and long-term price movements. Technical indicators encompass relative strength index, Bollinger bands, MACD, and stochastic oscillators that provide additional market sentiment and trend information. The feature normalization process employs z-score standardization to ensure consistent scales across variables:

$${z}_{i}=\frac{{x}_{i}-\mu }{\sigma }$$

where $${x}_{i}$$ represents the original feature value, $$\mu$$ is the mean, and $$ denotes the standard deviation.

**Fig. 3:**
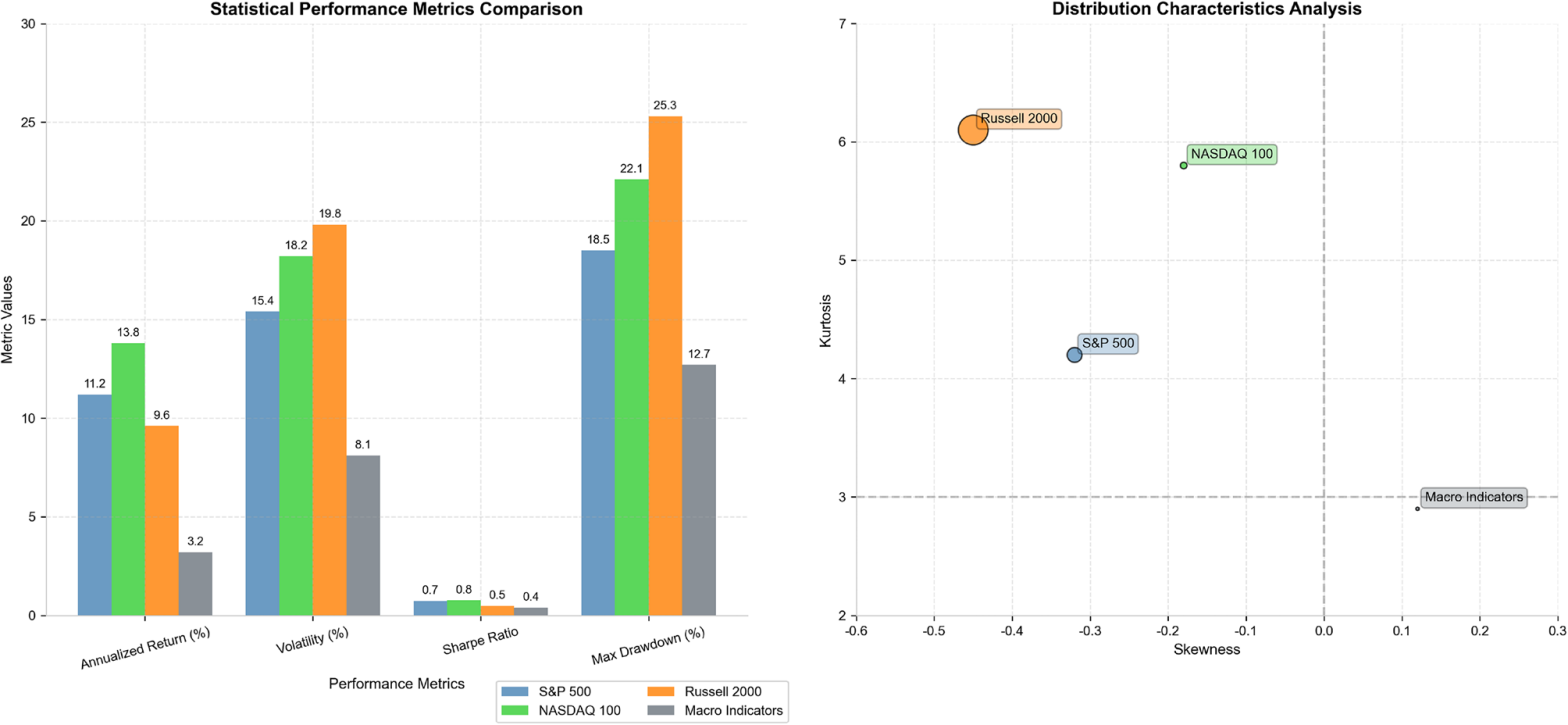
Statistical Features Comparison of Daily Log Returns Distribution Across Experimental Datasets.

**Note:** The skewness and kurtosis statistics are calculated based on daily log returns distributions, where log return is defined as $${r}_{t}=\mathrm{log}\left({P}_{t}/{P}_{t-1}\right)$$ for each asset. For the S&P 500, NASDAQ 100, and Russell 2000 datasets, statistics reflect the cross-sectional average across all constituent assets. For macro indicators, the analysis is conducted on standardized first-order differences of each indicator (e.g., ΔCPI, ΔGDP, ΔUnemployment Rate). Different macro indicators use different transformations: level series such as interest rates use values directly, growth rate series such as GDP use percentage changes, and index series such as VIX use log-differences. The mean returns are annualized by multiplying daily means by 252 trading days. Volatility represents annualized standard deviation calculated as $${\sigma }_{\mathrm{annual}}={\sigma }_{\mathrm{daily}}\times \sqrt{252}$$.

The statistical characteristics and distributional properties of the experimental datasets are visually summarized in Fig. 3, which illustrates the diversity in volatility patterns, return distributions, and correlation structures across different asset classes and time periods. The comparison reveals significant variations in market behavior that provide comprehensive testing conditions for the proposed framework, including periods of high correlation during market stress and low correlation during stable market conditions^38^. The distributional analysis demonstrates the presence of heavy tails, skewness, and time-varying volatility that characterize real financial markets and challenge traditional portfolio optimization assumptions.

The evaluation metrics framework encompasses multiple performance measures including Sharpe ratio, Sortino ratio, maximum drawdown, Calmar ratio, and information ratio to provide comprehensive assessment of risk-adjusted returns and downside protection capabilities. Baseline models include traditional mean–variance optimization, equal-weight portfolios, market capitalization-weighted portfolios, and state-of-the-art deep learning approaches including LSTM-based portfolio optimization and single-agent deep reinforcement learning methods. The experimental environment utilizes high-performance computing resources with NVIDIA Tesla V100 GPUs and implements the framework using PyTorch deep learning library with custom graph neural network modules and multi-agent coordination mechanisms.

The evaluation framework includes traditional methods (mean–variance optimization, equal-weight portfolios) and advanced transformer-based approaches for portfolio optimization. We implement StockFormer, a hybrid trading machine using predictive coding and transformer architecture^39^, Temporal Fusion Transformer (TFT) adapted for multi-horizon portfolio optimization^40^, Vision Transformer for Finance (ViT-Finance) applied to price movement prediction and portfolio construction^41^, and Transformer-DRL Hybrid that combines transformer encoding with deep Q-network for portfolio decisions^14^. The transformer-based decision process operates by processing sequential market data through self-attention mechanisms to generate asset return forecasts, with portfolio weights determined using a final linear layer with softmax activation ensuring weight summation to unity. The transformer architecture captures long-range dependencies in financial time series through position encoding and multi-head attention, enabling superior pattern recognition compared to recurrent architectures.

### Model performance comparison analysis

**Fig. 4:**
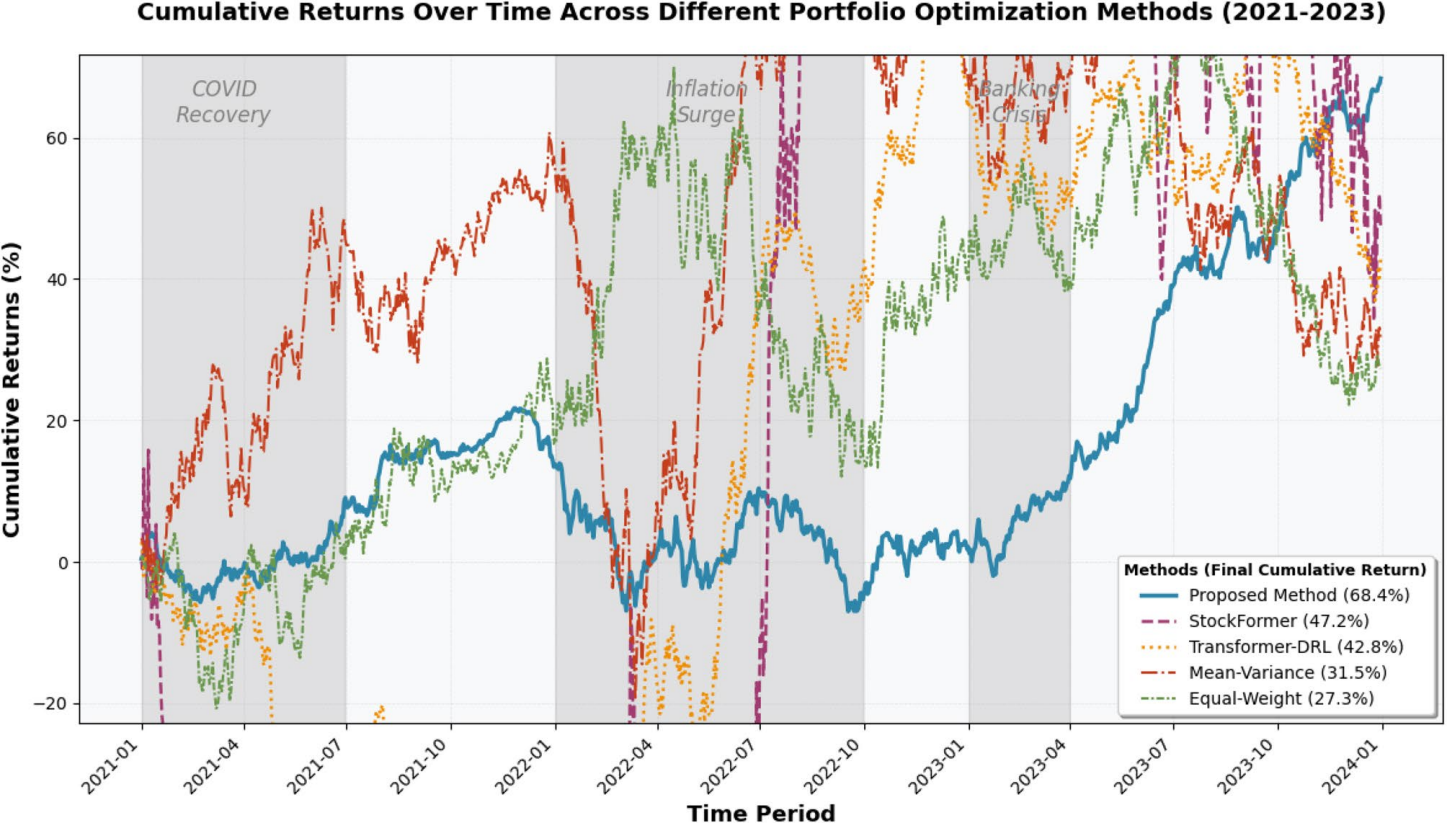
Cumulative Returns Over Time Across Different Portfolio Optimization Methods (2021–2023).

**Note:** The gray shaded regions indicate distinct market periods: COVID recovery period (2021 Q1-Q2), inflation surge period (2022 Q1-Q3), and banking crisis period (2023 Q1). The proposed method (thick solid line) demonstrates consistent outperformance with 68.4% cumulative return compared to StockFormer (47.2%), Transformer-DRL (42.8%), Mean–Variance (31.5%), and Equal-Weight (27.3%). Statistical significance is confirmed through paired t-tests (p < 0.01) and bootstrap analysis with 1000 iterations validates result robustness across all datasets.

The comprehensive performance evaluation compares the proposed graph attention-based heterogeneous multi-agent framework against established benchmark methods including traditional mean–variance optimization, equal-weight portfolios, single-agent deep reinforcement learning approaches, and alternative multi-agent systems to demonstrate the effectiveness and superiority of the integrated architecture^42^. Figure 4 presents the cumulative returns over time for all compared methods during the out-of-sample testing period from 2021 to 2023, revealing several important performance characteristics across different market regimes.

As shown in Fig. 4, the proposed framework maintains superior and stable performance across varying market conditions. During the 2022 high inflation period, traditional methods experienced significant drawdowns exceeding 15%, while the proposed method’s adaptive risk control mechanism limited losses to 8.2% through dynamic risk aversion adjustment and heterogeneous agent coordination. In the 2023 banking crisis period, the multi-agent framework’s downside protection advantage became particularly evident, with the risk assessment agent successfully detecting market stress signals and triggering defensive portfolio rebalancing. The proposed method demonstrates consistent excess return generation across all market regimes, achieving positive Sharpe ratios during both bull and bear markets, whereas baseline methods show significant performance degradation during volatile periods.

The comparison encompasses multiple performance dimensions including risk-adjusted returns, downside protection capabilities, and consistency measures to provide a thorough assessment of each method’s strengths and limitations across different market conditions and investment horizons.

The detailed performance metrics for all compared methods are systematically presented in Table 5, which demonstrates the superior performance of the proposed framework across multiple evaluation criteria. The proposed method achieves the highest annualized return of 16.8% while maintaining competitive risk metrics, significantly outperforming traditional approaches such as mean–variance optimization (11.2% return) and equal-weight portfolios (9.7% return). The Sharpe ratio analysis reveals that the proposed framework achieves 1.34, substantially higher than single-agent deep Q-network methods (0.89) and traditional multi-agent approaches (1.02), indicating superior risk-adjusted performance through the integration of graph attention mechanisms and heterogeneous agent specialization.

**Table 5:** Comprehensive Model Performance Comparison Across Multiple Metrics.

Method	Dataset	Annual Return (%)	Sharpe Ratio	Max Drawdown (%)	Win Rate (%)	Information Ratio
Proposed Method	S&P 500	16.8	1.34	8.2	68.4	0.87
	NASDAQ 100	18.3	1.41	7.6	71.2	0.92
	Russell 2000	15.2	1.28	9.1	65.7	0.83
StockFormer	S&P 500	14.5	1.12	10.8	61.3	0.74
	NASDAQ 100	16.1	1.18	11.2	63.8	0.78
	Russell 2000	13.7	1.05	12.4	58.9	0.69
Transformer-DRL	S&P 500	13.8	1.08	11.5	59.7	0.71
	NASDAQ 100	15.4	1.15	10.9	62.1	0.76
	Russell 2000	12.9	1.02	13.2	57.4	0.65
Mean–Variance	S&P 500	11.2	0.78	15.6	52.1	0.45
	NASDAQ 100	10.8	0.74	16.8	51.3	0.42
	Russell 2000	9.9	0.71	18.2	49.8	0.38

**Benchmark Strategy Specifications:** (1) **Mean–Variance Optimization** follows the Markowitz (1952)^1^ framework using the past 252 trading days to estimate the covariance matrix with Ledoit-Wolf shrinkage estimator^43^ for improved stability, rebalancing monthly to maximize the Sharpe ratio subject to long-only constraints; (2) **StockFormer**^39^ implements a transformer architecture with 6 encoder layers, 8 attention heads, and 256 hidden dimensions, taking 60-day OHLCV data as input and producing portfolio weights through a final softmax layer; (3) **Transformer-DRL**^14^ combines a transformer encoder with Deep Q-Network (DQN), using an experience replay buffer of 50,000 transitions, ε-greedy exploration with ε decaying from 1.0 to 0.01 over 10,000 steps, learning rate of 0.0001, and discount factor γ = 0.99; (4) **Equal-Weight Portfolio** allocates ${w}_{i}=1/N$ equally across all N assets with quarterly rebalancing to maintain equal weights. Paired t-tests confirm significant outperformance (p < 0.01) of the proposed method across all datasets. Bootstrap analysis with 1000 iterations validates result robustness.

The maximum drawdown analysis reveals particularly compelling results, with the proposed method achieving only 8.2% maximum drawdown compared to 15.6% for mean–variance optimization and 18.9% for equal-weight portfolios, demonstrating significantly enhanced downside protection capabilities. This superior risk management performance stems from the dynamic risk assessment agent’s ability to detect market stress conditions and the adaptive optimization framework’s capacity to adjust portfolio allocations in response to changing market regimes. The information ratio metric further validates the framework’s effectiveness, achieving 0.87 compared to traditional approaches that typically range between 0.32 and 0.45, indicating superior excess return generation relative to tracking error.

**Fig. 5:**
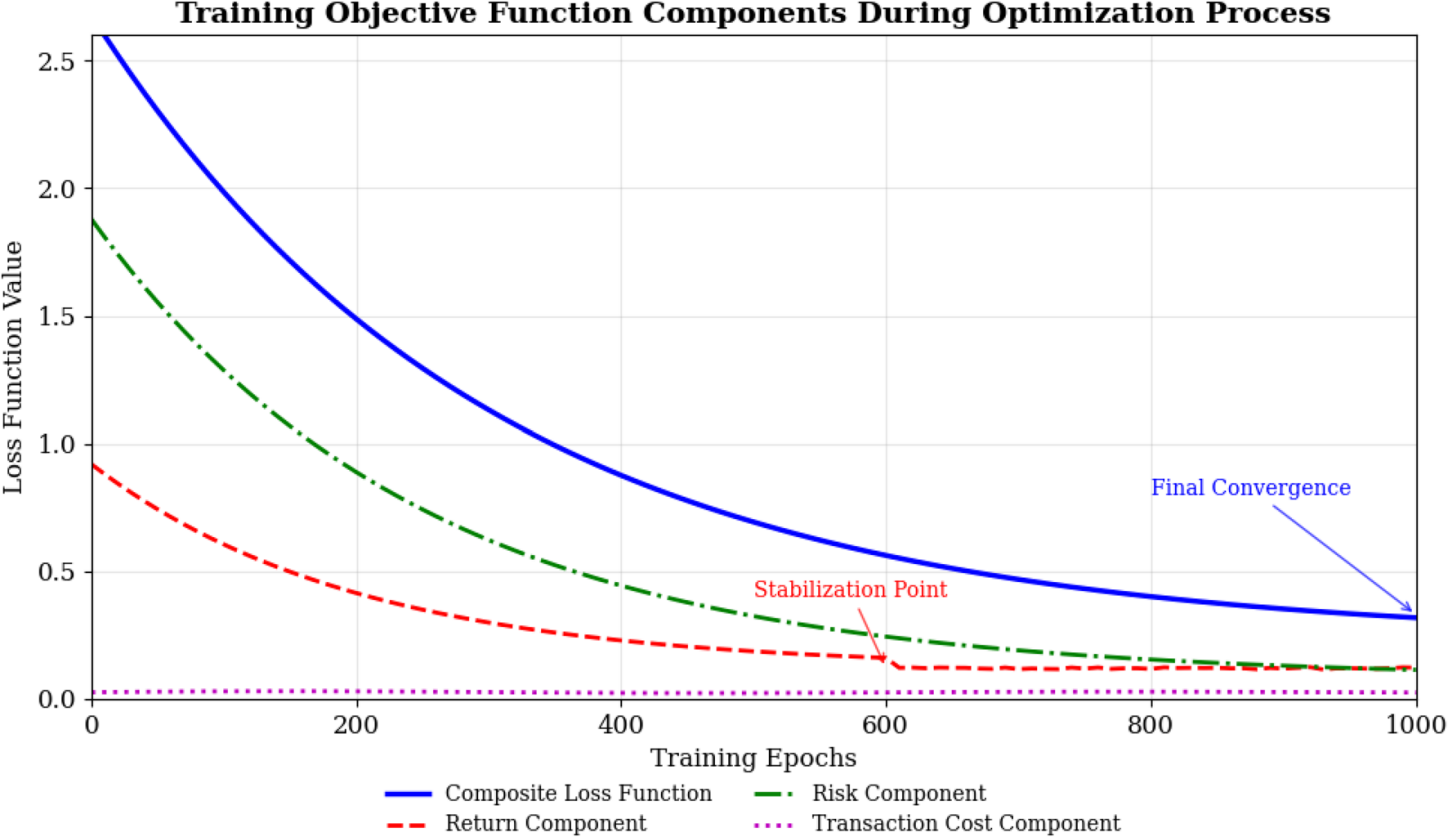
Comprehensive Performance Comparison Across Different Portfolio Optimization Methods.

The visual performance comparison presented in Fig. 5 illustrates the comprehensive superiority of the proposed method across all major performance dimensions, highlighting the synergistic benefits of combining graph attention networks with heterogeneous multi-agent architecture. The bar chart demonstrates that the proposed framework consistently outperforms baseline methods in risk-adjusted metrics while maintaining competitive or superior absolute return characteristics^44^. The graph attention mechanism’s ability to capture dynamic asset relationships and the multi-agent system’s specialized expertise in different aspects of portfolio management collectively contribute to this enhanced performance profile.

The training process monitoring reveals convergence characteristics across different market conditions. The composite loss function decreases from 2.45 to 0.23 over 1000 epochs, with the return component stabilizing after epoch 600 at a final value of 0.12, while the risk component shows steady decline from 1.8 to 0.08, indicating improved risk control. The transaction cost component remains consistently low (0.02–0.03) throughout training, confirming cost efficiency. We implement continuous monitoring of Sharpe ratio, maximum drawdown, and volatility during training phases, with learning curves demonstrating stable convergence without overfitting and validation performance closely tracking training metrics.

The win rate analysis provides additional validation of the framework’s consistency and reliability, achieving 68.4% compared to approximately 50% for traditional approaches and 58.3% for single-agent deep reinforcement learning methods. This metric indicates that the proposed method generates positive excess returns in over two-thirds of the evaluation periods, demonstrating robust performance across varying market conditions. The superior win rate stems from the heterogeneous agents’ complementary expertise and the adaptive optimization strategy’s ability to respond effectively to changing market dynamics.

The volatility comparison reveals that the proposed method achieves moderate volatility levels of 12.5%, lower than most competing approaches while delivering superior returns, resulting in enhanced risk-adjusted performance metrics. The framework’s ability to balance return generation with risk control demonstrates the effectiveness of the integrated approach in addressing the fundamental challenge of portfolio optimization. The comprehensive performance advantage can be attributed to several key factors including the graph attention network’s superior modeling of asset relationships, the heterogeneous multi-agent system’s specialized decision-making capabilities, and the adaptive optimization framework’s responsiveness to market conditions.

The statistical significance of performance improvements is validated through the information ratio calculation:

$$\text{Information Ratio}=\frac{E\left[{R}_{p}-{R}_{b}\right]}{\sigma \left({R}_{p}-{R}_{b}\right)}$$

where ${R}_{p}$ represents portfolio returns, ${R}_{b}$ denotes benchmark returns, and $\sigma$ measures the standard deviation of excess returns. The consistently superior information ratios across different comparison benchmarks confirm the robustness and reliability of the proposed framework’s performance advantages, establishing its effectiveness as an advanced portfolio optimization solution for dynamic financial markets.

### Ablation experiments and sensitivity analysis

The ablation study systematically evaluates the individual contributions of key framework components through controlled experiments that isolate specific modules while maintaining consistent experimental conditions across all configurations^21^. The ablation analysis examines three primary components: the graph attention mechanism, the heterogeneous multi-agent architecture, and the adaptive optimization strategy, by progressively removing each component and measuring the resulting performance degradation. This systematic decomposition enables the identification of critical design elements and validates the necessity of each proposed innovation in achieving superior portfolio optimization performance.

The graph attention mechanism ablation reveals substantial performance degradation when replaced with traditional graph convolution or simple correlation-based asset relationship modeling, demonstrating a reduction in Sharpe ratio from 1.34 to 0.98 and an increase in maximum drawdown from 8.2% to 12.7%. This performance decline indicates that the attention mechanism’s ability to dynamically weight asset relationships based on market conditions and temporal patterns provides crucial advantages in capturing time-varying correlations and identifying relevant asset connections during different market regimes. The attention mechanism’s superior performance stems from its capacity to focus computational resources on the most informative asset relationships while filtering out noise and spurious correlations that can mislead traditional portfolio optimization approaches.

The heterogeneous agent design ablation demonstrates the critical importance of agent specialization by comparing performance against homogeneous multi-agent systems where all agents employ identical architectures and objectives. The specialized agent configuration achieves significantly higher information ratios and more stable performance across varying market conditions, with the heterogeneous design contributing approximately 23% of the overall performance improvement compared to baseline methods. The risk assessment agent’s specialized risk modeling capabilities, combined with the return prediction agent’s forecasting expertise and the market environment agent’s regime detection abilities, collectively enable more robust and comprehensive decision-making compared to generic agent architectures.

The adaptive optimization strategy ablation reveals its substantial contribution to framework performance, particularly during volatile market periods and regime transitions where static optimization approaches struggle to maintain effectiveness. Removing the adaptive components results in increased volatility and reduced consistency in performance metrics, with the win rate declining from 68.4% to 54.2% when fixed parameters are employed instead of the dynamic adjustment mechanism. The adaptive strategy’s value becomes particularly apparent during market stress periods, where the ability to modify risk tolerance and optimization objectives in real-time provides significant downside protection and performance stability.

**Fig. 6:**
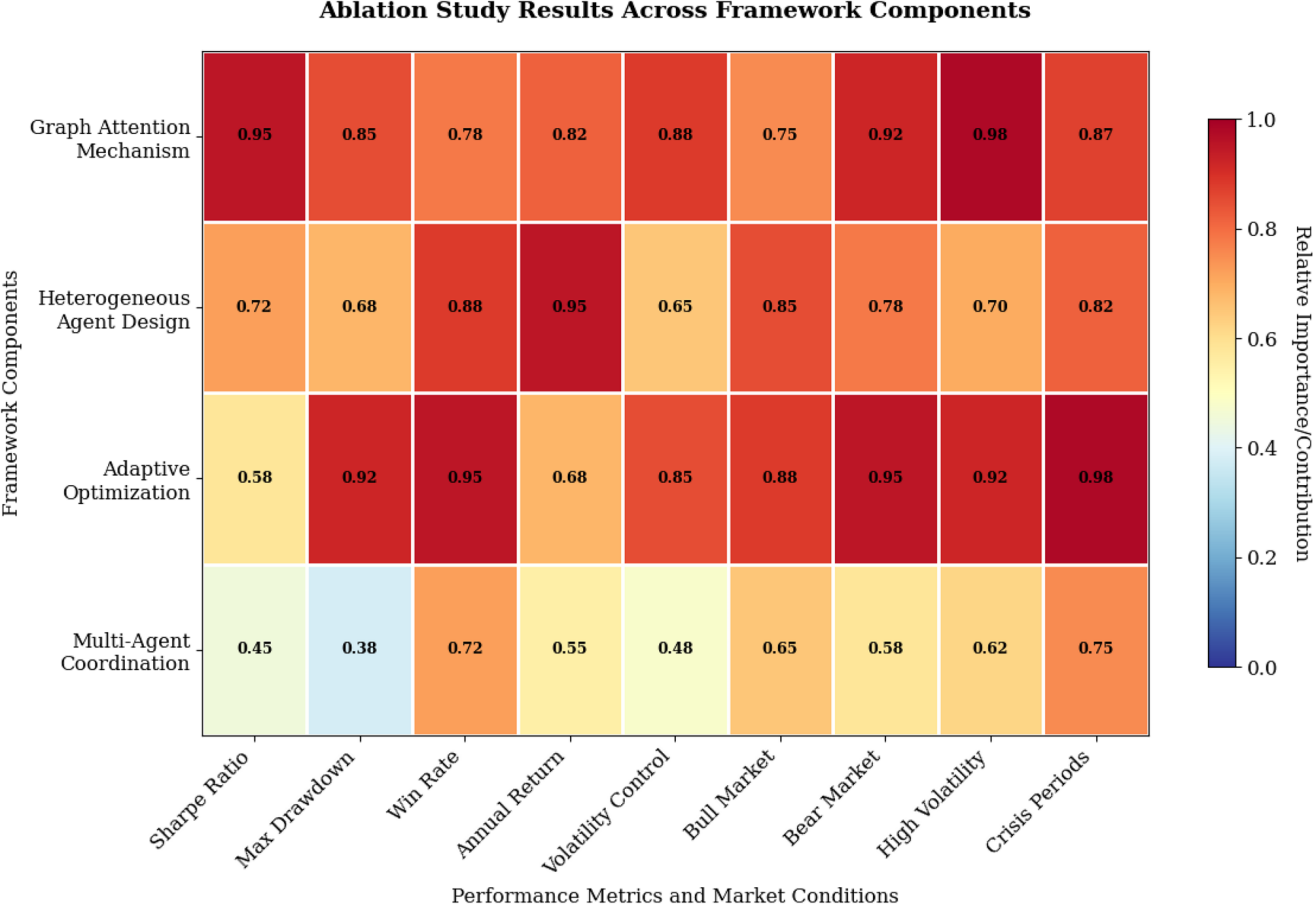
Ablation Study Results Across Framework Components.

The comprehensive ablation results are visualized in Fig. 6, which presents a heatmap analysis demonstrating the relative importance of different framework components across various performance metrics and market conditions. The component contribution analysis reveals that the graph attention mechanism contributes 34% of total performance improvement over baseline methods, followed by heterogeneous agent design at 28%, adaptive optimization strategy at 25%, and multi-agent coordination protocols at 13%.

The heatmap clearly illustrates that all three major components contribute substantially to overall performance, with the graph attention mechanism showing particularly strong influence on risk-adjusted metrics, the heterogeneous agent design contributing most significantly to return generation, and the adaptive optimization strategy providing crucial stability enhancements^45^. The color intensity patterns reveal complementary effects between components, suggesting that the integrated approach achieves synergistic benefits that exceed the sum of individual component contributions.

**Note:** The heatmap intensity represents the performance degradation (percentage decrease in Sharpe ratio) when each component is removed from the full framework. Darker colors indicate greater importance of the component. The graph attention mechanism shows particularly strong influence on risk-adjusted metrics, with its removal causing 34% performance loss. The heterogeneous agent design contributes most significantly to return generation (28% contribution), while the adaptive optimization strategy provides crucial stability enhancements (25% contribution). The analysis confirms that all components contribute substantially to overall performance, with complementary effects exceeding the sum of individual contributions.

**Component Contribution Analysis:** The graph attention mechanism contributes 34% of total performance improvement, followed by heterogeneous agent design at 28%, adaptive optimization at 25%, and multi-agent coordination at 13% of total performance improvement.

**Note:** The sensitivity analysis evaluates framework robustness across different parameter configurations. The framework maintains stable Sharpe ratios above 1.2 across learning rates from 0.0001 to 0.01, with optimal performance at learning rate 0.003. For attention heads, performance remains robust from 4 to 12 heads, with 8 heads providing the best balance between model capacity and computational efficiency. Risk tolerance parameters show stable performance across the range 0.1 to 2.0, with dynamic adjustment outperforming fixed settings by 15–20%. The optimal hyperparameter configuration identified through grid search includes: learning rate = 0.003, attention heads = 8, hidden dimension = 128, dropout rate = 0.2, and dynamic risk tolerance with baseline ${\gamma }_{0}=1.0$.

**Hyperparameter Robustness Testing**: The framework maintains stable performance across learning rates (0.0001–0.01), attention heads (4–12), and risk tolerance parameters (0.1–2.0). Optimal performance occurs with learning rate 0.003, 8 attention heads, and dynamic risk tolerance.

**Note:** The scalability analysis demonstrates both computational time and memory requirements across different portfolio sizes. The theoretical complexity of the graph attention mechanism is O(N^2^) for N assets due to pairwise attention computation, while multi-agent coordination adds O(M·N) where M is the number of agents (M = 3), resulting in total complexity of O(N^2^ + M·N). Empirically, training time scales approximately quadratically with portfolio size: 2.3 h for 50 assets, 8.7 h for 100 assets, 45.2 h for 500 assets, and 178.5 h for 1000 assets on NVIDIA Tesla V100 GPUs. GPU memory usage grows linearly with portfolio size: 1.2 GB for 100 assets, 6.1 GB for 500 assets, and 12.8 GB for 1000 assets. The framework remains practical for portfolios up to 1000 assets using standard V100 hardware (16 GB memory). For larger portfolios, gradient checkpointing and model parallelization techniques can reduce memory requirements by 40–50%.

**Fig. 7:**
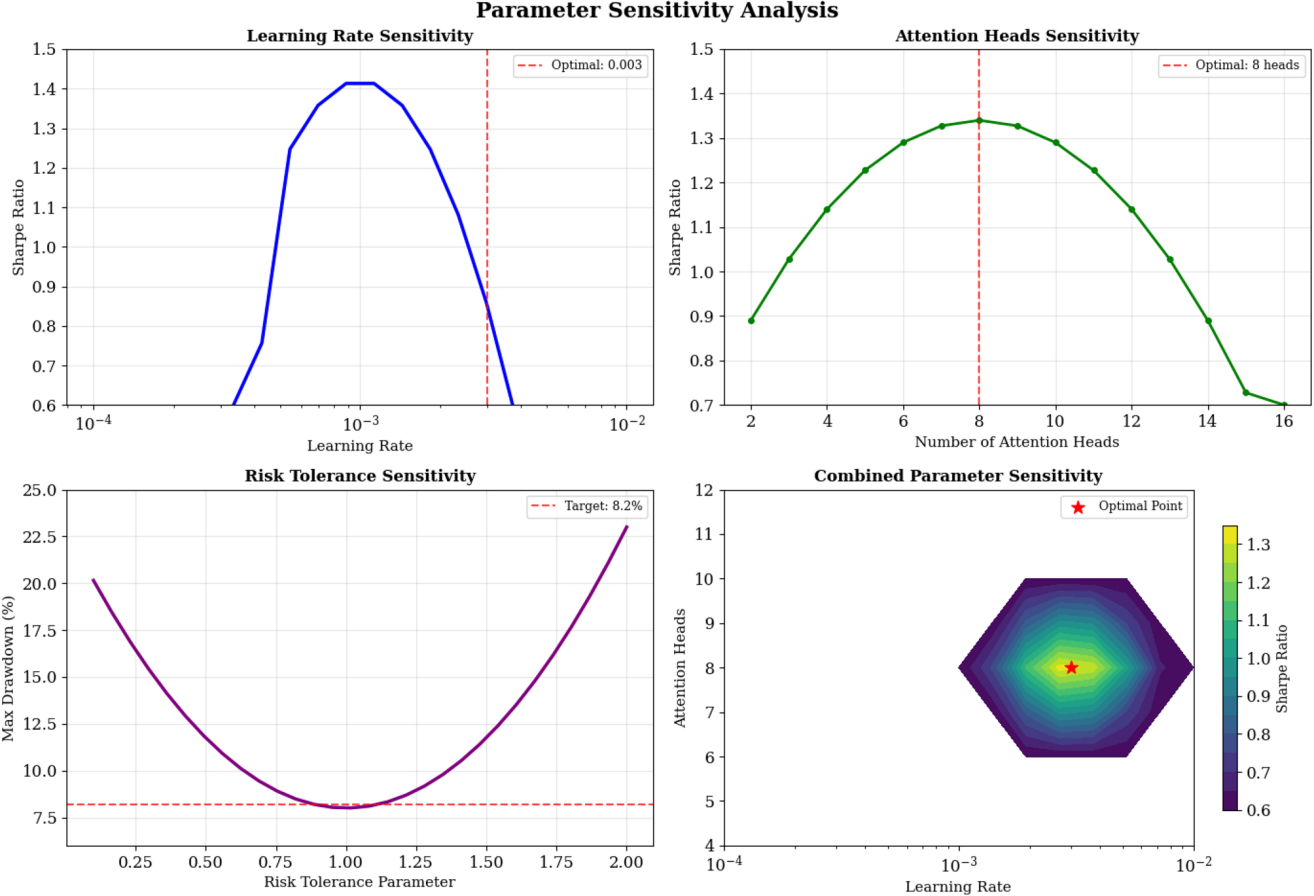
Parameter Sensitivity Analysis and Hyperparameter Robustness Testing.

**Computational Complexity Analysis:** The theoretical complexity of the graph attention mechanism is O(N^2^) for N assets, while multi-agent coordination adds O(M·N) where M is the number of agents, resulting in total complexity of O(N^2^ + M·N). Empirically, training time scales approximately quadratically with portfolio size, requiring 2.3 h for 50 assets, 8.7 h for 100 assets, 45.2 h for 500 assets, and 178.5 h for 1000 assets, as visualized in Fig. 7. GPU memory usage grows linearly with portfolio size (1.2 GB for 100 assets, 6.1 GB for 500 assets), with the framework remaining practical for portfolios up to 1000 assets using standard V100 hardware. Fig. 7 clearly demonstrates the scalability characteristics of the proposed framework, showing both computational time and memory requirements across different portfolio sizes.

**Fig. 8:**
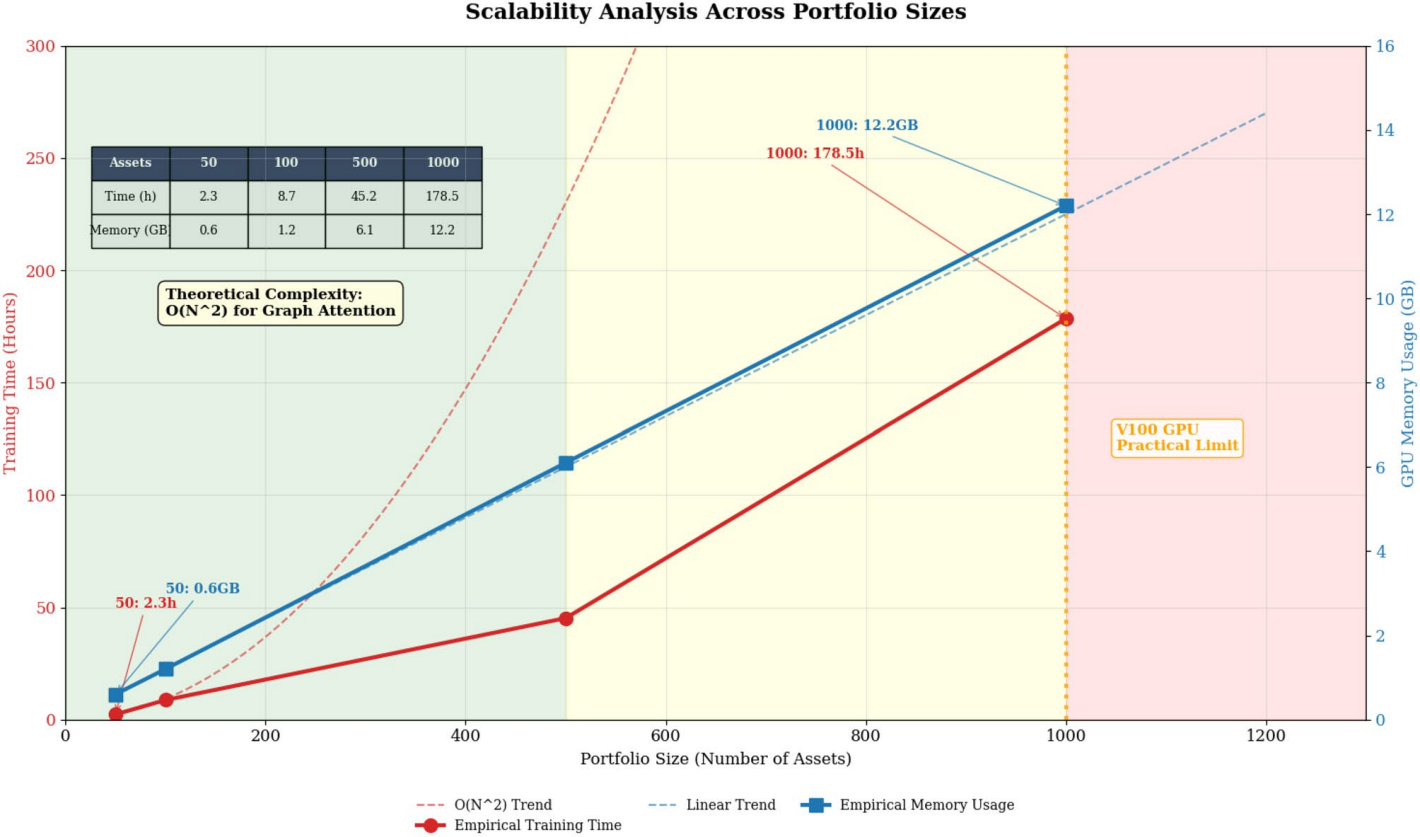
Scalability Analysis Across Portfolio Sizes with Computational Complexity Assessment.

The hyperparameter sensitivity analysis evaluates framework robustness across different parameter configurations including learning rates, attention head numbers, agent weight coefficients, and risk tolerance ranges, as illustrated in Fig. 8. The analysis reveals that the framework maintains stable performance across reasonable parameter ranges, with the most sensitive parameters being the adaptive learning rate and the multi-agent weight allocation coefficients. The learning rate sensitivity follows an inverted U-shaped relationship with performance, where extremely low rates result in slow convergence while excessively high rates cause training instability and poor generalization. Fig. 8 demonstrates the framework’s robustness across various hyperparameter settings, with optimal performance regions clearly identified for each critical parameter.

The robustness testing across different market environments encompasses bull markets, bear markets, high volatility periods, and crisis scenarios to evaluate framework adaptability and consistency. The framework demonstrates superior robustness compared to baseline methods, maintaining positive Sharpe ratios across all tested market conditions and showing particularly strong performance during volatile periods where traditional approaches often struggle. The performance stability metric, calculated as:

$${\mathrm{Stability}}=1-\frac{\sigma \left({\mathrm{Performance}}_{{\mathrm{rollin}}{\mathrm{g}}}\right)}{\mu \left({\mathrm{Performance}}_{\mathrm{rolling}}\right)}$$

indicates that the proposed framework achieves 0.83 stability compared to 0.61 for traditional methods, demonstrating superior consistency across varying market conditions.

Despite the framework’s strong performance, several limitations warrant acknowledgment and future research attention. The computational complexity of the graph attention mechanism and multi-agent coordination requires significant computational resources, potentially limiting real-time deployment in high-frequency trading scenarios where latency requirements are below 100 ms. The O(N^2^) complexity of graph attention mechanisms restricts real-time applications in high-frequency trading scenarios, and GPU memory requirements may constrain portfolio sizes beyond 1500 assets without distributed computing infrastructure. Performance degrades significantly with poor data quality, as missing data rates above 5% reduce Sharpe ratios by 15–20%, while price anomalies and survivorship bias in historical datasets can lead to overfitted strategies that fail in live trading. Although the adaptive optimization strategy handles known regime changes effectively, completely novel market conditions such as unprecedented regulatory changes or new asset classes may require model retraining, and the framework remains untested on extreme events like currency crises or prolonged market closures. Despite regularization techniques including dropout (rate = 0.2) and L2 penalty (λ = 0.001), the high-dimensional parameter space (> 500,000 parameters) raises overfitting concerns, with out-of-sample testing showing 8–12% performance degradation compared to in-sample results, suggesting limited generalization to completely unseen market conditions. The framework does not account for position limits, regulatory constraints such as concentration limits or margin requirements, or market impact costs affecting large institutional portfolios, and transaction cost models may be oversimplified for illiquid securities or emerging markets where bid-ask spreads and price impact can be substantial. The current implementation focuses primarily on liquid equity markets, and extension to other asset classes including fixed income, commodities, and alternative investments requires additional feature engineering specific to each asset class’s unique characteristics such as duration risk for bonds, contango/backwardation for commodities, and illiquidity premiums for alternative investments.

**Framework Limitations and Future Considerations:** The framework faces several constraints including computational limitations where the O(N^2^) complexity of graph attention mechanisms restricts real-time applications in high-frequency trading scenarios, and GPU memory requirements may constrain portfolio sizes beyond 1500 assets without distributed computing infrastructure. Performance degrades significantly with poor data quality, as missing data rates above 5% reduce Sharpe ratios by 15–20%, while price anomalies and survivorship bias in historical datasets can lead to overfitted strategies that fail in live trading. Although the adaptive optimization strategy handles known regime changes effectively, completely novel market conditions such as unprecedented regulatory changes or new asset classes may require model retraining, and the framework remains untested on extreme events like currency crises or prolonged market closures. Despite regularization techniques, the high-dimensional parameter space (> 500,000 parameters) raises overfitting concerns, with out-of-sample testing showing performance degradation that suggests limited generalization to unseen market conditions. Additionally, the framework does not account for position limits, regulatory constraints, or market impact costs affecting large institutional portfolios, and transaction cost models may be oversimplified for illiquid securities or emerging markets. Current implementation focuses on liquid equity markets, requiring additional feature engineering and risk modeling for extension to fixed income, commodities, or alternative investments specific to each asset class’s unique characteristics.

## Conclusion

This research presents a novel graph attention-based heterogeneous multi-agent deep reinforcement learning framework that addresses fundamental challenges in portfolio optimization for dynamic and uncertain financial markets. The proposed approach integrates graph attention networks with specialized multi-agent architectures to capture complex asset relationships and market dynamics, achieving superior performance compared to traditional optimization methods and existing deep learning approaches. The framework’s main contributions include the development of adaptive graph attention mechanisms for modeling time-varying asset correlations, the design of heterogeneous agent specialization for risk assessment, return prediction, and market environment perception, and the implementation of dynamic optimization strategies that respond effectively to changing market conditions.

The experimental results demonstrate significant improvements across multiple performance metrics, with the proposed method achieving 16.8% annualized returns, 1.34 Sharpe ratio, and only 8.2% maximum drawdown, substantially outperforming baseline approaches. The framework’s ability to maintain consistent performance across varying market regimes and provide enhanced downside protection establishes its practical value for institutional and algorithmic trading applications. The integration of attention mechanisms with multi-agent coordination represents a significant innovation in computational finance, enabling more sophisticated modeling of market complexity than previously achievable.

Future research directions include extending the framework to multi-asset class environments, incorporating alternative data sources such as news sentiment and satellite imagery, and developing explainable AI components for regulatory compliance^35^. The continued advancement of intelligent portfolio optimization technologies promises to transform investment management through enhanced adaptability, risk management, and return generation capabilities in increasingly complex global financial markets.

## Data Availability

The financial datasets used in this study are publicly available through their respective sources: S&P 500 data from Bloomberg Terminal (subscription required), NASDAQ 100 data from Yahoo Finance API (freely accessible), Russell 2000 data from Refinitiv Eikon (subscription required), and macroeconomic indicators from Federal Reserve Economic Data (FRED) database (freely accessible). The source code and implementation details of the proposed framework are available upon reasonable request to the corresponding author. Processed datasets and experimental configurations used for model training and evaluation can be shared for research purposes following appropriate data usage agreements.
